# SNP Microarray in FISH Negative Clinically Suspected 22q11.2 Microdeletion Syndrome

**DOI:** 10.1155/2016/5826431

**Published:** 2016-03-09

**Authors:** Ashutosh Halder, Manish Jain, Amanpreet Kaur Kalsi

**Affiliations:** Reproductive Biology, AIIMS, New Delhi 110029, India

## Abstract

The present study evaluated the role of SNP microarray in 101 cases of clinically suspected FISH negative (noninformative/normal) 22q11.2 microdeletion syndrome. SNP microarray was carried out using 300 K HumanCytoSNP-12 BeadChip array or CytoScan 750 K array. SNP microarray identified 8 cases of 22q11.2 microdeletions and/or microduplications in addition to cases of chromosomal abnormalities and other pathogenic/likely pathogenic CNVs. Clinically suspected specific deletions (22q11.2) were detectable in approximately 8% of cases by SNP microarray, mostly from FISH noninformative cases. This study also identified several LOH/AOH loci with known and well-defined UPD (uniparental disomy) disorders. In conclusion, this study suggests more strict clinical criteria for FISH analysis. However, if clinical criteria are few or doubtful, in particular newborn/neonate in intensive care, SNP microarray should be the first screening test to be ordered. FISH is ideal test for detecting mosaicism, screening family members, and prenatal diagnosis in proven families.

## 1. Introduction

The 22q11.2 microdeletion syndrome is the most common microdeletion syndrome and seen at a prevalence of 1 in 4000 to 6000 live births [1]. It is characterized by hemizygous microdeletion of ≤3 mb size of chromosome 22q11.2 locus in which several genes are lost. It is mostly spontaneous/de novo and in <10% cases are inherited [2–4]. It is frequently associated with multiple congenital anomalies, in particular cardiac anomaly (conotruncal cardiac anomaly such as Fallot's tetralogy, interrupted aortic arch, truncus arteriosus, or major aortopulmonary collateral), developmental delay, hypocalcaemia, immune deficiency, cleft palate or velopharyngeal insufficiency or swallowing difficulty, and dysmorphism (broad bulbous nose, square shaped tip of nose, short philtrum, telecanthus, slanting eyes, low set ears, etc.). FISH, until recent, was commonly used for precise genetic diagnosis of common microdeletion syndromes, including 22q11.2 microdeletion [5–8]. However, FISH provides information only on targeted locations and does not allow a comprehensive evaluation of the whole genome. In addition, atypical smaller deletions are difficult to identify by FISH due to failure in covering those locations by single FISH probe (outside the region of hybridisation by the FISH probe). Furthermore, it is difficult to detect 22q11.2 duplication by FISH due to variable size of signals as well as distraction with split signals of normal cells. Often 22q11.2 duplication displays features like 22q11.2 deletion syndrome [9]. Hence, FISH alone cannot provide reliable diagnosis for cases of 22q11.2 microdeletions/duplications syndrome. Further, if FISH is used for doubtful cases (if few numbers of clinical criteria are fulfilled by patient) then chances of detecting targeted deletion are less frequent [7, 8]. High-resolution array CGH was used to investigate FISH negative for 22q11.2 deletions cases with conotruncal heart defects [10] and additional cases of 22q11.2 microdeletion/microduplication containing TBX1 gene were detected. In this study with SNP microarray we have assessed 101 cases of FISH negative/noninformative clinically suspected 22q11.2 microdeletion syndrome to determine whether any benefit is gained from using SNP microarray.

## 2. Material and Methods

This study was conducted in the Department of Reproduction Biology, All India Institute of Medical Sciences, New Delhi, India, from September 2011 to August 2014. A total of 101 FISH negative/noninformative (FISH was normal in 85 cases, was not attempted in 12 cases due to frozen/clotted samples, and failed in 4 cases due to few/lysed/clumped cells) clinically suspected 22q11.2 microdeletion syndrome cases were prospectively enrolled for the study using SNP microarray. FISH was carried out using noncommercial FISH probe (22q11.2; RP5-882J5; genomic coordinate/exact position of probe is unavailable; obtained from Uniba Biologia, University of Bari, Italy, by curtsy of Professor Mariano Rocchi, http://www.biologia.uniba.it/rmc/). SNP microarray was used to detect chromosomal abnormality (aneuploidy, triploidy, mosaicism, etc.) as well as microdeletion, microduplication, UPD/LOH, and so forth as described before [11]. All cases were referred from various hospitals of Delhi (northern India) for confirmation of clinical diagnosis. The Institutional Human Ethics Committee approved research protocol. Most patients (excluding very sick/intensive care patients) underwent clinical genetics evaluation (directly by our team or by referral physician) as per specific 22q11.2 microdeletion syndrome proforma (Supplementary file 1 in Supplementary Material available online at http://dx.doi.org/10.1155/2016/5826431). About 2 mL EDTA blood sample was collected from the affected individual. SNP microarray study was carried out commercially either from Illumina (HumanCytoSNP-12 DNA Analysis Bead Chip Kit 300 K) or Affymetrix (GeneChip Human Mapping 750 K) platforms. Images were captured on iScan System or Cytoscan, respectively. Data were analyzed (primary) using Illumina's KaryoStudio/GenomeStudio and Affymetrix ChAS software. Resolution was set as 0.1 mb for deletion, 0.5 mb for gain, and 3 mb for LOH/AOH. Secondary analysis was carried out using web data analysis resources like DECIPHER (GRCH37; version 9.4), OMIM, and UPD with cross reference to UCSC, NCBI, Ensembl, and DGV. Interpretation and reporting followed American College of Medical Genetics standards and guidelines for interpretation and reporting of postnatal constitutional copy number variants [12].

## 3. Result

A total of 101 DNA samples from FISH negative/noninformative clinically suspected 22q11.2 microdeletion syndrome were analyzed by SNP microarray successfully. Details of SNP microarray findings of all 101 FISH negative/noninformative samples are available as master table (Supplementary file 2). Out of 101 FISH negative for 22q11.2 microdeletion cases, SNP microarray detected several cases of chromosomal abnormalities (Table 1), 22q11.2 microdeletion/microduplication (Table 2), other microdeletion/microduplication cases (Table 3), and several cases of likely pathogenic CNVs (Table 4). SNP microarray also detected several cases of LOH/AOH of known UPD disorders (Table 5) and some more likely pathogenic UPD disorders (Table 6) in the study.

**Figure 1:**
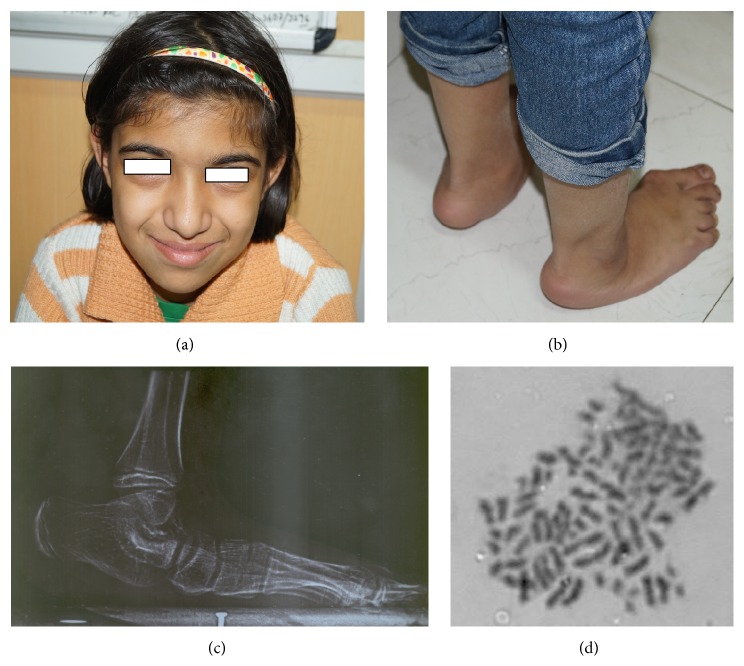
Mosaic triploidy case showing facial profile (a), flat rocker bottom foot ((b) clinical and (c) X-ray), and triploid metaphase cell (d).

**Figure 2:**
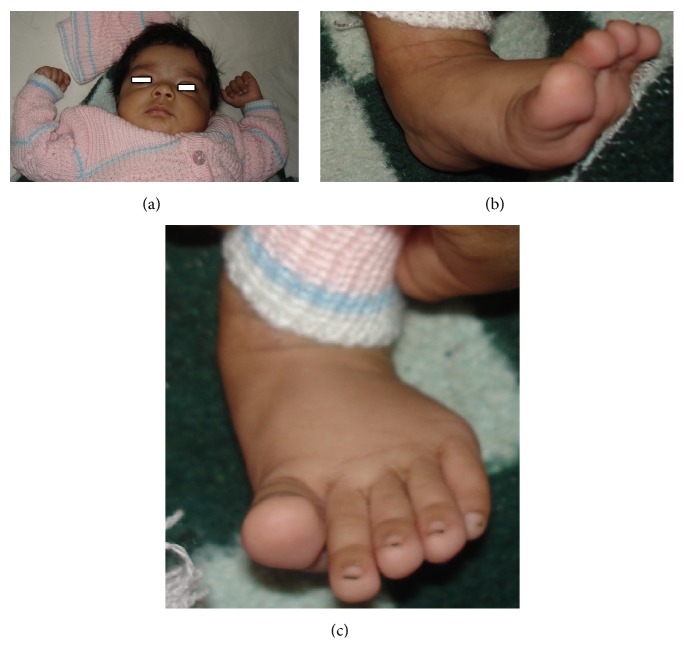
Male child with 15q11.2 deletion and 10q11.22 duplication showing squint, small toe and deep furrow feet.

**Figure 3:**
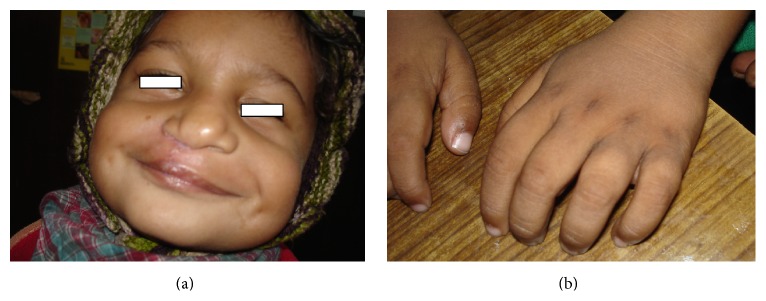
Male child with LOH/AOH of 6p22.3-p21.32 showing repaired cleft lip, broad and bifid nose, small left cornea and eye, long fingers, and rough creased skin over hands.

This SNP microarray study identified 6 cases of chromosomal abnormalities (Table 1). Clinical feature in brief is presented in Table 7 (Figure 1). The study also identified eight cases of 22q11.2 microdeletions (7)/microduplication (1), mostly from FISH noninformative cases and 2 smaller CNVs (outside scope of FISH probe). Clinical feature in brief is presented in Table 8. This study detected 4 cases of other pathogenic CNVs (Table 3) and several cases of likely pathogenic CNVs (Table 4), which were outside the scope of FISH. Clinical features in brief of these cases are presented in Tables 9 and 10 (Figure 2). Microarray analysis identified 9 cases of segmental LOH/AOH, those associated with known UPD disorders (Table 5). Clinical features in brief of these cases are presented in Table 11 (Figure 3).

## 4. Discussion

We have been investigating microdeletion syndrome using FISH since 2005. Our experience with FISH in microdeletion syndrome including 22q11.2 microdeletion syndrome (most common microdeletion syndrome) is unsatisfactory as FISH detects approximately 8% of cases of 22q11.2 microdeletions [7, 8]. The reasons for low positive rate of 22q11.2 microdeletion syndrome using RP5-882J5 PAC clone (FISH probe for 22q11.2 locus) were poor clinical inclusion criteria (often single criteria) and poor quality of sample referral (clotted/frozen/clumped blood samples/cells leading to FISH failure or omission of FISH tests). In our experience (8, 11–13), FISH probe derived from RP5-882J5 PAC clone identifies all typical deletions (≥2 mb); however, it is unable to detect atypical deletions of ≤0.5 mb sizes (at proximal or distal LCRs). Number of diagnostic criteria fulfilled by our patients ranged from one to eight and most consistent referral criteria were broad nose, TOF, swallowing difficulty, and hypocalcaemia/convulsion. Patient often was referred to our laboratory for 22q11.2 FISH testing based on a single clinical feature, namely, CHD (TOF) or convulsion. These lead to lower deletion detection rate. Poirsier et al. [13] also observed poor clinical criteria for FISH test referral.

In this prospective study, SNP microarray was carried out in 101 FISH negative/noninformative clinically suspected 22q11.2 microdeletion syndrome cases to assess the role of SNP microarray in the evaluation of clinically suspected 22q11.2 microdeletion syndrome. There were 12 samples that were not processed for FISH due to freezing or clotting. Microarray was successfully carried out in all 101 cases. Chen et al. [10], like this study, investigated FISH negative 22q11.2 deletion cases using high-resolution array CGH. They have found better diagnostic sensitivity of array CGH over FISH in fetuses with cardiac abnormalities associated with deletion 22q11.2 and duplication 22q11.2 syndromes. In the present study we have also detected several cases of chromosomal abnormalities (trisomy, triploidy, partial monosomy, or partial trisomy) and other pathogenic/likely pathogenic CNVs (Tables 1, 3, and 4). We have also picked up some smaller specific deletions/duplications outside the region of hybridisation of the FISH probe. Clinically suspected specific CNV (22q11.2) was detectable in approximately 8% of cases by SNP microarray over and above FISH, mostly from FISH failure samples (frozen/clotted samples; FISH not tried). In fact, it was unexpected from FISH to identify small atypical deletions outside the region of hybridization in two cases (database numbers 48 and 191), which were identified by microarray. Thus, microarray contributed actual improvement by ~2% above FISH for specific 22q11.2 deletions. However, SNP microarray provided many other pathogenic/likely pathogenic microdeletions/duplications besides aneuploidy/partial aneuploidy, triploidy, UPD, and other disorders. We have also observed poor clinical criteria as the leading contributor of failure to detect specific 22q11.2 deletion. Variations in deletion size and/or break point difference (with genes involvement) as well as other CNVs with or without LOH were evident. This study also identified several cases of LOH/AOH loci with known and well-defined UPD disorders. Several cases of suspected LOH/AOH were also identified; however, confirmation of association needs additional investigation using SNP microarray/QF PCR of their parents before claiming causative.

One of the major referral criteria for our patients was congenital heart disease, mainly conotruncal heart defect [7]. Several studies have established the importance of 22q11.2 CNVs in the etiology of congenital heart disease (CHD) with or without other associated malformations [14, 15]. Patients with 22q11.2 duplication also present clinical features like 22q11.2 deletion syndrome, including heart defect [16]. In 22q11.2 duplication syndrome, most prevalent heart defect was conotruncal heart defect [17]. This is also true in animal experiment where mice carrying extra copies of TBX1 gene display full clinical picture of 22q11.2 deletion syndrome [18]. In this study we have found 2 cases of atypical small CNVs of 22q11.2 (duplication of 0.18 mb spanning 18844632–19033532 and containing genes like DGCR6, PRODH, KIAA1647, DGCR9, DGCR10, and DGCR2 and deletion of 0.38 mb spanning 19405537–19792353 and containing genes like UFD1L, PI4KA, SERPIND1, SNAP29, CRKL, AIFM3, AIFM3, LZTR1, THAP7, FLJ39582, MGC16703, P2RX6, P2RX6, SLC7A4, P2RX6P, LOC400891, and TBX1) with multiple malformations including cardiac malformations. These atypical small deletions are rarely reported [13, 19]. The role of TBX1 in heart development has been already demonstrated in mice [20] and TBX1 mutations have also been identified in individuals with TOF [21]. We have also observed TBX1 deletion with TOF, in our series.

This study detected several cases of LOH/AOH of known UPD disorders (Table 5) and some more likely pathogenic UPDs (Table 6); however, further study using SNP microarray on parental DNA is required before reporting associations with specific developmental disorders.

Our study indicates that microarray should be first tier of test when samples are scant, lysed, or clumped/clotted/frozen as FISH or conventional cytogenetics are bound to fail/noninformative. This study suggests that microarray is a superior technique in clinically doubtful cases as well as ICU admissions on life support and 22q11.2 microdeletion is suspected on the basis of convulsion and/or cardiac defect/failure. Furthermore, in early weeks of life dysmorphology and malformation detection is difficult, in particular sick neonate on life support. We have observed in this study that clinically suspected microdeletion syndrome cases are frequently associated with second/more hits (deletion or duplication) elsewhere in the genome (tables/associated CNVs). Microarray also detected several cases of chromosomal aneuploidy, partial aneuploidy, triploidy, and so forth. This approach of DNA microarray will provide the highest chance of making a diagnosis and sparing the patient unnecessary diagnostic testing from many places, in addition to saving crucial times. Our conclusion is in agreement with the consensus statement [22].

We conclude that more strict clinical criteria should be followed for FISH test. If clinical diagnosis is uncertain or doubtful then microarray should be the first screening test. This is most important with newborn/neonate in intensive care unit as clinical criteria are few and difficult to elicit. Microarray is applicable in all samples irrespective of frozen, lysed, clotted, clumped, and other samples. Furthermore, SNP microarray provides information on aneuploidy, triploidy, partial aneuploidy, and associated small CNVs (often many) besides information on LOH/AOH (indicating UPD disorders/in case of consanguinity homozygosity of recessive disorders). FISH may be used for detecting mosaicism, screening family members, prenatal diagnosis, and preimplantation diagnosis of specific deletions in proven family.

## Supplementary Material

Supplementary file 1: Clinical proforma for evaluation of suspected 22q11.2 micro-deletion (also provides useful guideline for clinical diagnosis)
Supplementary file 2: Master table of the study showing details of FISH and SNP microarray findings of all 101 FISH negative/non-informative samples

## Figures and Tables

**Table 1:** Details of chromosomal abnormalities in FISH negative^*∗*^ suspected 22q11.2 microdeletion cases.

DBN	Locus/loci	CNV	Start	End	Size (mb)	Genes	Disease
93	18p11.32-p11.21	3	2842	15365878	15.3	89	Trisomy 18
	18q11.1-q23	3	16786754	76115554	59.3	241	
98	All chromosomes	3					Triploidy (mosaicism confirmed by FISH)
103	18q21.31-q23	1	54144903	78014582	23.8		Partial 18q monosomy
115	11q23.2-q25	3	113077541	134944006	21.8		Partial 11q trisomy (Jacobsen, Bartter 2, 11q)
116	All chromosomes	3					Triploidy (mosaicism confirmed by FISH)
205	21q11.2 q21.1	3	13609442	46923252	33.3	256	Trisomy 21
	q21.2 q21.3						
	q22.11 q22.12						
	q22.13 q22.2						
	q22.3						

FISH negative^*∗*^ means normal FISH result or FISH that failed or FISH not carried out due to cell lysis (frozen sample) or clotted sample.

DBN: database number (CNV 1 = deletion; CNV 3 = duplication).

**Table 2:** Details of 22q11.2 microdeletion and/or microduplication in FISH negative^*∗*^ suspected 22q11.2 microdeletion cases.

DBN	FISH	Locus	CNV	Start	End	Size (mb)	Genes (no)	Gene details
42	Not done due to frozen blood sample	22q11.21	1	18877787	21914652	3	58	DGCR6; PRODH; KIAA1647; DGCR9; DGCR10; DGCR2; DGCR2; DGCR14; TSSK2; GSC2; SLC25A1; CLTCL1; HIRA; MRPL40; C22orf39; C22orf39; UFD1L; CDC45L; CLDN5; LOC150185; SEPT5; GP1BB; TBX1; GNB1L; C22orf29; TXNRD2; COMT; COMT; COMT; COMT; ARVCF; C22orf25; MIR185; DGCR8; MIR1306; TRMT2A; RANBP1; ZDHHC8; Em:AC006547.7; RTN4R; MIR1286; DGCR6L; LOC375133; RIMBP3; ZNF74; SCARF2; KLHL22; MED15; POM121-like 1; DKFZp434N035; PI4KA; SERPIND1; SNAP29; CRKL; AIFM3; AIFM3; LZTR1; THAP7; FLJ39582; MGC16703; P2RX6; P2RX6; SLC7A4; P2RX6P; LOC400891

47	Not done due to frozen blood sample	22q11.21	1	17257787	19792353	2.53	68	DGCR6; PRODH; KIAA1647; DGCR9; DGCR10; DGCR2; DGCR11; DGCR14; TSSK2; GSC2; SLC25A1; CLTCL1; HIRA; MRPL40; C22orf39; C22orf39; UFD1L; CDC45L; CLDN5; LOC150185; SEPT5; GP1BB; TBX1; GNB1L; C22orf29; TXNRD2; COMT; COMT; COMT; COMT; ARVCF; C22orf25; MIR185; DGCR8; MIR1306; TRMT2A; RANBP1; ZDHHC8; LOC150197; RTN4R; MIR1286; DGCR6L; LOC375133; RIMBP3; ZNF74; SCARF2; KLHL22; MED15; POM121L4P; TMEM191A; PI4KA; SERPIND1; SNAP29; CRKL; AIFM3; AIFM3; LZTR1; THAP7; FLJ39582; MGC16703; P2RX6; P2RX6; SLC7A4; P2RX6P; LOC400891

48	Not done due to frozen blood sample	22q11.21	1	19405537	19792353	0.38	16	PI4KA; SERPIND1; SNAP29; CRKL; AIFM3; AIFM3; LZTR1; THAP7; FLJ39582; MGC16703; P2RX6; P2RX6; SLC7A4; P2RX6P; LOC400891; TBX1

87	Not done due to sample clotting	22q11.21	1	18877787	21050552	2.1	47	DGCR6; PRODH; KIAA1647; DGCR9; DGCR10; DGCR2; DGCR2; DGCR14; TSSK2; GSC2; SLC25A1; CLTCL1; HIRA; MRPL40; C22orf39; C22orf39; UFD1L; CDC45L; CLDN5; LOC150185; SEPT5; GP1BB; TBX1; GNB1L; C22orf29; TXNRD2; COMT; COMT; COMT; COMT; ARVCF; C22orf25; MIR185; DGCR8; MIR1306; TRMT2A; RANBP1; ZDHHC8; Em:AC006547.7; RTN4R; MIR1286; DGCR6L; LOC375133; RIMBP3; ZNF74; SCARF2; KLHL22; MED15; POM121-like 1

182	Not done due to sample clotting	22q11.21	1	18877787	21811991	2.9	69	DGCR6; PRODH; KIAA1647; DGCR9; DGCR10; DGCR2; DGCR2; DGCR14; TSSK2; GSC2; SLC25A1; CLTCL1; HIRA; MRPL40; C22orf39; C22orf39; UFD1L; CDC45L; CLDN5; LOC150185; SEPT5; GP1BB; TBX1; GNB1L; C22orf29; TXNRD2; COMT; COMT; COMT; COMT; ARVCF; C22orf25; MIR185; DGCR8; MIR1306; TRMT2A; RANBP1; ZDHHC8; Em:AC006547.7; RTN4R; MIR1286; DGCR6L; LOC375133; RIMBP3; ZNF74; SCARF2; KLHL22; MED15; POM121-like 1; DKFZp434N035; PI4KA; SERPIND1; SNAP29; CRKL; AIFM3; AIFM3; LZTR1; THAP7; FLJ39582; MGC16703; P2RX6; P2RX6; SLC7A4; P2RX6P; LOC400891; POM121L8P; RIMBP3C; RIMBP3B; HIC2

188	Not done due to sample clotting	22q11.21	1	18877787	21798907	2.9	69	DGCR6; PRODH; KIAA1647; DGCR9; DGCR10; DGCR2; DGCR2; DGCR14; TSSK2; GSC2; SLC25A1; CLTCL1; HIRA; MRPL40; C22orf39; C22orf39; UFD1L; CDC45L; CLDN5; LOC150185; SEPT5; GP1BB; TBX1; GNB1L; C22orf29; TXNRD2; COMT; COMT; COMT; COMT; ARVCF; C22orf25; MIR185; DGCR8; MIR1306; TRMT2A; RANBP1; ZDHHC8; Em:AC006547.7; RTN4R; MIR1286; DGCR6L; LOC375133; RIMBP3; ZNF74; SCARF2; KLHL22; MED15; POM121-like 1; DKFZp434N035; PI4KA; SERPIND1; SNAP29; CRKL; AIFM3; AIFM3; LZTR1; THAP7; FLJ39582; MGC16703; P2RX6; P2RX6; SLC7A4; P2RX6P; LOC400891; POM121L8P; RIMBP3C; RIMBP3B; HIC2

191	Normal	22q11.21	3	18844632	19033532	0.18	6	DGCR6; PRODH; KIAA1647; DGCR9; DGCR10; DGCR2

244	Not done due to sample clotting	22q11.21	1	19004730	21800471	2.79	68	DGCR9, DGCR10, DGCR2, DGCR11, DGCR14, TSSK2, GSC2, SLC25A1, CLTCL1, HIRA, MRPL40, C22orf39, UFD1L, CDC45, CLDN5, LINC00895, SEPT5, SEPT5-GP1BB, GP1BB, TBX1, GNB1L, C22orf29, TXNRD2, COMT, MIR4761, ARVCF, TANGO2, MIR185, DGCR8, MIR3618, MIR1306, TRMT2A, RANBP1, ZDHHC8, LOC388849, LOC284865, LINC00896, RTN4R, MIR1286, DGCR6L, LOC729444, TMEM191B, PI4KAP1, RIMBP3, ZNF74, SCARF2, KLHL22, MED15, POM121L4P, TMEM191A, PI4KA, SERPIND1, SNAP29, CRKL, AIFM3, LZTR1, THAP7, THAP7-AS1, TUBA3FP, P2RX6, SLC7A4, P2RX6P, LOC400891, BCRP2, POM121L8P, RIMBP3C, RIMBP3B, HIC2

FISH negative^*∗*^ means normal FISH result or FISH that failed or FISH not carried out due to cell lysis (frozen sample) or clotted sample.

DBN: database number (CNV 1 = deletion; CNV 3 = duplication).

**Table 3:** Details of other microdeletions and/or duplications in FISH negative^*∗*^ suspected 22q11.2 microdeletion cases.

DBN	Locus/loci	CNV	Start	End	Size	Genes	Gene details/disease
88	15q11.2	3	22754322	23222284	0.46	6	Prader-Willi/Angelman syndrome and chromosome 15q11-q13 duplication syndrome (TUBGCP5; CYFIP1; CYFIP1; NIPA2; NIPA1; WHAMML1)

102	16p11.2	1	29661217	30347731	0.68	46	C16orf54; MAZ; PRRT2; C16orf53; MVP; MVP; CDIPT; LOC440356; LOC440356; SEZ6L2; ASPHD1; KCTD13; TMEM219; TAOK2; HIRIP3; INO80E; DOC2A; FLJ25404; FAM57B; ALDOA; ALDOA; ALDOA; ALDOA; PPP4C; TBX6; YPEL3; YPEL3; GDPD3; MAPK3; LOC100271831; CORO1A; LOC606724; BOLA2; GIYD1; SULT1A4; SULT1A4; LOC388242; LOC613037; IMAA; CD2BP2; TBC1D10B; MYLPF; SEPT1; ZNF48; ZNF771; DCTPP1

197	1q21.1	3	144943150	146916824	1.9	24	PDZK1; GPR89A; GPR89C; PDZK1; LOC200030; NBPF11; LOC728989; PRKAB2; PDIA3P; FMO5; FMO5; CHD1L; BCL9; ACP6; GJA5; GJA8; GPR89B; GPR89C; PDZK1; LOC200030; NBPF11; FLJ39739; PPIAL4B; PPIAL4A; NBPF14; PPIAL4F; NBPF15; NBPF15; NBPF16; PPIAL4E; NBPF16; PPIAL4F; LOC645166; LOC645166

229	15q11.1	3	20192086	22508324	2.3	13	GOLGA6L6; GOLGA8C; BCL8; POTEB; NF1P1; LOC646214; CXADR; POTEB; NF1P1; LOC727924; OR4M2; OR4N4; OR4N3P
	q11.2						

FISH negative^*∗*^ means normal FISH result or FISH that failed or FISH not carried out due to cell lysis (frozen sample) or clotted sample.

DBN: database number (CNV 1 = deletion; CNV 3 = duplication).

**Table 4:** Details of likely pathogenic CNVs in FISH negative^*∗*^ suspected 22q11.2 microdeletion cases.

DBN	Locus/loci	CNV	Start	End	Size	Genes	Gene details/disease
18	22q11.22	3	22901370	23307965	0.4	7	PRAME, LOC648691, POM121L1P, GGTLC2, MIR650, IGLL5; hsa-mir-650
	7q11.21	1	64651296	65148399	0.49	3	INTS4L1, ZNF92, INTS4L2 (cutis marmorata, epicanthus, everted lower lip vermilion, intellectual disability, microcephaly, retinoblastoma, short nasal septum, and thick lower lip vermilion)

20	1q21.2	3	147828939	149723885	1.9	18	2-3-toe syndactyly, abnormality of the helix, aplasia cutis congenita over the scalp vertex, attention deficit hyperactivity disorder, clinodactyly of the 5th finger, facial asymmetry, flat occiput, gait ataxia, global developmental delay, hyperpigmentation of the skin, hypoplastic areola, long eyelashes, microcephaly, muscular hypotonia, pointed chin, short 3rd toe, short neck, short stature, skull asymmetry, and walking on tiptoes FLJ39739; PPIAL4B; PPIAL4A; NBPF14; PPIAL4F; NBPF15; NBPF15; NBPF16; PPIAL4E; NBPF16; PPIAL4F; LOC645166; LOC645166; LOC388692; FCGR1C; HIST2H2BF; PPIAL4B; LOC728855

46	19p13.11 p12 p11	3	19863014	24556461	4.6	35	LOC284440; ZNF506; ZNF253; ZNF93; ZNF682; ZNF90; ZNF486; LOC284441; ZNF826; ZNF737; ZNF626; ZNF626; ZNF85; ZNF430; ZNF714; ZNF431; ZNF708; ZNF738; ZNF493; ZNF429; ZNF100; LOC641367; ZNF43; ZNF208; ZNF257; ZNF676; ZNF98; ZNF492; ZNF99; ZNF91; ZNF675; ZNF681; RPSAP58; ZNF254; LOC100101266
	11p11.2 p11.12	3	48359268	51530241	3.1	10	OR4C45; OR4A47; FOLH1; LOC440040; OR4C13; OR4C12; LOC441601; LOC646813; OR4A5; OR4C46

49	1q31.3	4	193973521	197781198	3.8	13	KCNT2; CFH; CFHR3; CFHR1; CFHR4; CFHR2; CFHR5; F13B; ASPM; ZBTB41; CRB1; DENND1B; DENND1B
	Xq21.1-q22.1	2	80368850	99441815	19	27	HMGN5; SH3BGRL; POU3F4; CYLC1; RPS6KA6; HDX; UBE2DNL; APOOL; SATL1; ZNF711; POF1B; CHM; CHM; DACH2; DACH2; KLHL4; CPXCR1; TGIF2LX; PABPC5; PCDH11X; PCDH11X; PCDH11X; NAP1L3; FAM133A; LOC643486; DIAPH2; RPA4; LOC442459
	Xq23 q24	2	112244679	117105172	4.8	18	HTR2C; SNORA35; MIR764; MIR1912; MIR1264; MIR1298; MIR1911; MIR448; IL13RA2; LRCH2; RBMXL3; LUZP4; PLS3; PLS3; AGTR2; SLC6A14; CXorf61; KLHL13
	Xq27.2-q28	2	141247401	148041505	6.7	36	MAGEC2; SPANXN4; SPANXN3; SLITRK4; SPANXN2; UBE2NL; SPANXN1; SLITRK2; SLITRK2; CXorf1; MIR890; MIR888; MIR892A; MIR892B; MIR891B; MIR891A; CXorf51; CXorf51; MIR506; MIR507; MIR508; MIR509-2; MIR509-3; MIR509-3; MIR509-1; MIR509-2; MIR509-3; MIR510; MIR514-1; MIR514-3; MIR514-3; ASFMR1; FMR1; FMR1NB; AFF2; AFF2

51	1q21.1	3	144007842	145384225	1.3	12	LOC728855; LOC728875; PPIAL4B; PPIAL4A; LOC728875; COAS3; NBPF20; PDE4DIP; PDE4DIP; PDE4DIP; SEC22B; NOTCH2NL; NBPF10
	Xp22.33	2	449065	1705775	1.2	10	SHOX; CRLF2; CRLF2; CSF2RA; CSF2RA; IL3RA; SLC25A6; PP1164; ASMTL; P2RY8

71	15q11.2	1	20482360	22754322	2.2	19	CYFIP1; CYFIP1; p; WHAMML1; GOLGA9P; HERC2P2; GOLGA8E; MKRN3; MAGEL2; NDN; PWRN2; PWRN1; C15orf2; SNRPN; SNRPN; SNURF; SNURF
	10q11.22	3	46167847	48338944	2.1	23	BMS1P1; FAM35B; SYT15; SYT15; GPRIN2; PPYR1; LOC728643; ANXA8; ANXA8L1; FAM25C; LOC642826; FAM35B2; ANTXRL; ANXA8L2; FAM21B; LOC642826; FAM25G; ANXA8; ANXA8L1; ZNF488; RBP3; GDF2; GDF10

197	1q21.1	3	144943150	146916824	1.9	24	PDZK1; GPR89A; GPR89C; PDZK1; LOC200030; NBPF11; LOC728989; PRKAB2; PDIA3P; FMO5; FMO5; CHD1L; BCL9; ACP6; GJA5; GJA8; GPR89B; GPR89C; PDZK1; LOC200030; NBPF11; FLJ39739; PPIAL4B; PPIAL4A; NBPF14; PPIAL4F; NBPF15; NBPF15; NBPF16; PPIAL4E; NBPF16; PPIAL4F; LOC645166; LOC645166

204	22q11.23	3	23980648	24240879	0.26	2	IGLL3; LRP5L (downslanted palpebral fissures, long palpebral fissure, moderate global developmental delay, short upturned nose, and wide nasal bridge)

FISH negative^*∗*^ means normal FISH results or FISH that failed or FISH not carried out due to cell lysis (frozen sample) or clotted sample.

DBN: database number (CNV 1 = deletion; CNV 3 = duplication).

**Table 5:** Details of LOH/AOH detected with known UPD (uniparental disomy) disorder in suspected 22q11.2 microdeletion.

DBN	Cytoband	Size (mb)	Gene number	UPD disorder	Genes	Remarks (associated CNVs and LOH/AOH)
17	11q13.4-q14.1	12.3	96	Dysmorphism and mental retardation	Unknown	One benign (3) CNV of 8 (p23.3-p23.2) Two LOH/AOH of chromosome 11

45	14q32.2	3.0	11	Kagami-Ogata syndrome/Temple syndrome	DLK1, GTL2, RTL1, and so forth	No CNV No other LOH/AOH

75	6p22.3-p22.1	7.4	112	Diabetes mellitus, transient neonatal, 1	ZFP57	No CNV No other LOH/AOH
	p21.33 p21.32					

83	7p12.2-p11.2	7.3	29	Silver-Russell syndrome (7p11.2-p13)	GRB10	One benign (3) CNV (20q13.33) Multiple LOH/AOH of other chromosomes

97	6q24.2-q25.1	8.2	49	Transient neonatal diabetes mellitus and isolated cleft lip and palate	PLAGL1	Two uncertain (1) CNVs (15q11.2) One likely benign (0) CNV (10q22.3) One LOH/AOH of 16p13.11-p12.3

110	7q31.32-q35	20	167	Silver-Russell syndrome (7q31-qter)	MEST	One likely benign (3) CNV (15q11.2) Multiple LOH/AOH of chromosome 7 and other chromosomes

128	11p15.4	4.1	552	Beckwith-Wiedemann syndrome		No CNV Multiple LOH/AOH of same/other chromosomes

220	11p15.5-p15.4	3.5		Beckwith-Wiedemann syndrome	CDKN1C	One benign (1) CNV 19p12-p11 Multiple LOH/AOH of other chromosomes

227a	15q11.2 q12	4.8	701	Prader-Willi/Angelman syndrome	SNRPN, SNORD116, UBE3A	No associated CNVs Multiple LOH/AOH of other chromosomes

227b	11q24.2 q24.3	5.9	901	Dysmorphism and mental retardation	Unknown	No associated CNVs Multiple LOH/AOH of other/same chromosomes
	q25					

DBN: database number.

**Table 6:** Large (>5 mb) UPD/LOH/AOH locus/loci detected in suspected 22q11.2 microdeletion^*∗*^.

DBN	Cytoband	Size (mb)	Gene number	Remarks (associated CNVs and LOH/AOH)
20	6p21.1 p12.3	5.7	38	One uncertain (3) CNV of chromosome 1 (q21.2) Multiple LOH/AOH of chromosomes 6 and 1

43	4q21.23-q22.1	8.7	38	One benign (1) CNV of chromosome 9 (p24.3) One LOH/AOH of chromosome 15

49	Xq21.1 q21.2 q21.31	19	28	Partial tetrasomy of chromosomes 1–8, 11–14, and 18 and partial disomy of chromosome X
	q21.32 q21.33 q22.1			
	Xq27.2 q27.3 q28	6.7	25	Multiple LOH/AOH of chromosome X and one LOH/AOH of chromosome 1

73	2p25.2 p25.1	5.7	33	One likely benign (3) CNV of chromosome 4 (q22.3) Multiple LOH/AOH of many chromosomes
	2q21.1-q23.3	18	44	
	3p21.31-p14.2	13	161	
	3q21.1-3 q23	16	135	
	4q31.3-q35.1	32	112	
	6p21.1 p12.3	5.7	86	
	9p24.2-p23	8.3	29	
	10q25.1-q26.11	11.8	60	
	10q26.11-q26.3	12.8	68	
	11p15.4-p15.1	11.7	102	
	14q11.2 q12	8.8	63	
	14q12-q22.3	23.9	112	
	15q22.33-q25.2	14.4	155	
	18p11.31-p11.21	11.9	55	
	18q11.1-q12.3	22.8	85	

76	17q12-q21.1	5.3	102	One benign (1) CNV of chromosome 14 (q11.2) Multiple LOH/AOH of many chromosomes

84	3p21.31-p21.1	7	34	No CNVs; multiple LOH/AOH of many chromosomes

85	8q23.1 q23.2 q23.3	6	14	No CNVs No other LOH/AOH

87	6q21 q22.1 q22.2 q22.31	12	60	No CNVs One LOH/AOH of chromosome 11

101	6q14.1-q15	10.5	51	One benign CNV

105	1q23.1-q31.1	29.5	232	Multiple benign/likely benign CNVs Multiple LOH/AOH of many chromosomes
	3q27.1-q29	13.6	108	
	5q33.3-q35.3	24.7	195	
	7p14.2 p14.1	5.3	22	
	10p15.1 p14	5.4	34	
	16q22.3-q23.3	10	44	
	17p13.1-p11.2	11.7	124	
	18q12.1-q12.3	13	46	

112	5p15.2 p15.1	7	18	One benign CNV LOH/AOH of chromosome 11 (p11.2 p11.12)
	9q31.1-q32	8.3	71	

169	1p13.3-p12	9.9	101	2 likely benign CNVs of chromosomes 8 and X Multiple LOH/AOH of many chromosomes
	5q35.1-q35.3	8	107	
	10q11.21 q11.22	5.4	49	
	10q21.1-q22.1	19	81	
	10q24.1-q25.2	14	133	
	12q22-q24.32	34.3	281	
	14q23.3-q32.2	30.3	231	
	19q13.32-q13.42	7.2	272	
	20q13.13-q13.32	8	43	
	22q11.1-q12.1	11.4	173	
	Xp22.32-p21.1	27	123	
	Xq23 q24	9.8	51	

178	5p13.3 p13.2	5.8	37	No CNV
	17p11.2-q11.2	9.9	125	No other LOH/AOH

182	13q14.2-q21.32	18.6	75	No CNV No other LOH/AOH
	6p22.3-p21.33	10.3	269	
	2q32.3 q33.1	5.2	717	
	2q34-q36.1	9.6	765	
	2q36.1-q37.1	7.4	622	
	7p22.2-p21.3	5.7	49	

189	17q21.32-q23.2	13.3	175	No CNV

200	4q32.3-q34.3	9.7	616	No CNV 2 other LOH/AOH
	2q11.1-q12.1	8.8	996	
	2p12-p11.2	8.2	844	
	2p22.3 p22.2 p22.1	6.1	719	
	3p26.1 p25.3 p25.2 p25.1	7.8	830	
	15q23-q24.3	8	1205	

204	3q11.2 q12.1 q12.2 q12.3	6	49	One likely benign CNV No other LOH/AOH
	16p12.1 p11.2 p11.1	8	107	
	16q11.2 q12.1 q12.2	7	40	

211	5q21.3-q22.3	7.3	30	No CNV
	14q22.1-q23.1	7.3	51	One LOH/AOH of chromosome 7

214	18q11.2-q12.1	5.6	29	One likely benign CNV
	6p25.2-p24.3	5.5	42	Two LOH/AOH of chromosomes 12 and 19

228	8q24.21 q24.22	5.5	20	No CNV or no other LOH/AOH

^*∗*^To investigate its clinical importance/significance/associations/and so forth there is a need for further study using SNP microarray of parents.

DBN: database number.

**Table 7:** Clinical details of chromosomal abnormalities detected by SNP microarray.

Locus/loci (CNV)	Microarray diagnosis	Clinical details	Referral reason
18p11.32-p11.21 18q11.1-q23 (3)	Trisomy 18	Seven-month-old male with acyanotic congenital heart defect (CHD)	CHD To exclude 22q11.2 microdeletion

All chromosomes (3)	Triploidy (mosaicism confirmed by FISH; 33% triploid cells)	Five-year-old female (at first visit) and now 13 years old without any secondary sex character (Figure 1 **)**, less fetal movement during pregnancy, cesarean section delivery due to nonprogress of labor, feeding problem, DTGV (dextrotransposition of great vessels) operated and ASD (atrial septal defect), global developmental delay (GDD), broad thumb and toe, convulsion (controlled), hypertelorism, bulbus nose, rocker bottom feet, and periventricular leukomalacia on MRI	CHD To exclude 22q11.2 microdeletion

18q21.31-q23 (1)	Partial 18q monosomy	One-month-old male in intensive care unit (ICU), acyanotic CHD with congestive cardiac failure (CCF), and Noonan syndrome like face, plagiocephaly, low set ears, upslanted eyes, sandal gap, and so forth	?Noonan syndrome

11q23.2-q25 (3)	Partial 11q trisomy (Jacobsen, Bartter 2, 11q)	Seven-day-old female neonate in ICU, asymmetric IUGR (intrauterine growth restriction), preterm, multiple malformations, respiratory infection, VSD (ventricular septal defect), hypoplastic nail and alae nasi, blepharophimosis, hypotonia, and so forth	CHD To exclude 22q11.2 microdeletion

All chromosomes (3)	Triploidy (mosaicism confirmed by FISH; 24% triploid cells)	Eight-year-old female with dysmorphism (broad nose, cleft palate, thin lip, and long philtrum), long slender fingers, GDD, recurrent respiratory infection, behavioral problem, and so forth	Broad nose, behavioral problem To exclude 22q11.2 microdeletion

21q11.2 q22.3 (3)	Trisomy 21	Three-month-old male in Pediatric ICU with CCF, very sick	?CHD To exclude 22q11.2 microdeletion

**Table 8:** Clinical details of 22q11.2 CNVs detected by SNP microarray.

Microarray details	Genes	TBX1/DGCR2/DGCR6/DGCR14/DGCR8	Clinical details	Other CNVs
3 mb deletion	58	Y/Y/Y/Y/Y	Male of 3+ years with tetralogy of Fallot (TOF; operated) and facial dysmorphism, broad nose, feeding difficulty, and so forth	Nil

2.5 mb deletion	65	Y/Y/Y/Y/Y	7-year-old female with TOF and dysmorphism	7q11.21 (3)

0.38 mb deletion	15	Y/N/N/N/N	1.5-year-old male with TOF	Nil

2.17 mb deletion	49	Y/Y/Y/Y/Y	34-year-old male referred from anesthesia ICU for hypoparathyroidism, hypocalcemia, recurrent fungal infection, seizure, and respiratory failure (since last 45 days)	Nil

2.9 mb deletion	69	Y/Y/Y/Y/Y	1.5-year-old male with TOF, facial dysmorphism, GDD, and speech delay	Nil

2.9 mb deletion	69	Y/Y/Y/Y/Y	45-day-old female with TOF, recurrent intractable seizure, recurrent infection, suckling difficulties, low calcium, absent thymic shadow on X-ray, and history of polyhydramnios during pregnancy	Nil

0.18 mb duplication	6	N/Y/Y/N/N	10-month-old female with CHD, frontal bossing, prominent metopic suture, hypertelorism, V shaped lip, dysplastic ear, wide spaced nipple, pectus carinatum, mid gut volvulus, and so forth	2p22.3 (3) 11p11.12 (1) 14q11.2 (3)

2.79 mb deletion	68	Y/Y/Y/Y/Y	3-year-old male with seizure, GDD, dysmorphism, high arched palate, long slender fingers, low parathyroid hormone (PTH), low calcium, recurrent infection, and so forth	Nil

**Table 9:** Clinical details of other pathogenic CNVs detected by SNP microarray.

Microarray details	Genes	Diagnosis	Clinical details	Other CNVs
15q11.2 0.46 mb duplication	6	15q11-q13 duplication	Three-month-old male with TOF, feeding difficulty, hypocalcemia, dysmorphism, poly/syndactyle, hypoplastic mandible, IUGR, and so forth	10q11.22 (3)

16p11.2 0.68 mb deletion	46	16p11.2 deletion	4-month-male with CHD (DORV, PS/pulmonary stenosis), blepharophimosis, ptosis, and so forth	15q12 (3) 14q11.2 (3)

1q21.1 1.9 mb duplication	24	1q21.1 duplication	Male of 2+ years with TOF, broad nose, thin upper lip, absent philtrum, small and low set ears, antimongoloid slant, telecanthus, long slender fingers, widow peak, and so forth	11p11.12 (3)

15q11.1 q11.2 2.3 mb duplication	13	15q11.1 q11.2 duplication	1-year-old male with CHD (VSD), dysmorphism, GDD, and so forth	14q11.2 (3)

**Table 10:** Clinical details of likely pathogenic CNVs detected by SNP microarray.

Locus/loci	Microarray details	Genes	Clinical details
22q11.22 7q11.21	0.4 mb duplication 0.49 mb deletion	7 3	2-year-old male with TOF

1q21.2	1.9 mb duplication	18	9-year-old male with TOF (operated)

19p13.11-12 11p11.2	4.6 mb duplication 3.1 mb duplication	35 10	17-year-old female with TOF (operated)

1q31.3 Xq21.1-24 Xq27.2-28	3.8 mb triplication 23.8 mb duplication 6.7 mb duplication	13 27 18	13-year-old male with TOF (operated)

1q21.1 Xp22.33	1.3 mb duplication 1.2 mb duplication	12 10	4-year-old male with TOF (operated)

15q11.2 10q11.22	2.2 mb deletion 2.1 mb duplication	19 23	One-month-old male with hypocalcaemia (Ca 5.6; PTH-46), convulsion, osteopenia, squint, small toe, deep furrow feet, and so forth (Figure 2)

22q11.23	0.26 mb duplication	2	6-year-old female with dysmorphism, square nose tip, proportionate short stature, cyanotic CHD (tricuspid atresia, ostium secundum ASD, etc.), clubbing, tracheal shift, right pneumothorax with lung collapse, right anotia, synophrys, pear shaped nose, webbed neck, and so forth

**Table 11:** Clinical details of LOH/AOH detected by SNP microarray with known UPD disorders.

Locus/loci	Genes	Clinical details	UPD disorders
11q13.4-q14.1	96	9-year-old male with TOF (operated)	Dysmorphism and mental retardation

14q32.2	11	5-year-old male with TOF (operated)	Kagami-Ogata syndrome/Temple syndrome

6p22.3-p21.32	112	4-year-old male with TOF (operated), cleft lip and palate (repaired), broad and bifid nose, small left cornea and eye, GDD, hearing problem, being still unable to suck/drink, long fingers, small philtrum, mental retardation, and so forth (Figure 3)	Diabetes mellitus, transient neonatal, 1

7p12.2-p11.2	29	3.5-year-old male with TOF (operated), GDD, and previous 2 siblings with CHD	Silver-Russell syndrome (7p11.2-p13)

6q24.2-q25.1	49	10-month-old male with cyanotic CHD, GDD, obesity, hypospadias, no cryptorchidism, dysmorphism, and one elder sister who has CHD	Transient neonatal diabetes mellitus and isolated cleft lip and palate

7q31.32-q35	167	14-month-old female with TOF, small nose, wide philtrum, narrow forehead, low frontal hairline, GDD, broad nose, short stature, bilateral cataract, and previous 3 siblings with malformations (including CHD in one)	Silver-Russell syndrome (7q31-qter)

11p15.4	552	1.5-year-old male with cyanotic CHD, dysmorphism, clubbing, dysplastic small low set ears, weight of 8 kg (overweight), and so forth	Beckwith-Wiedemann syndrome

11p15.5-p15.4		6-day-old very sick (in ICU) male with dysmorphism, CCF, and so forth	Beckwith-Wiedemann

15q11.2-q12	701	9-year-old male with TOF (operated)	Prader-Willi/Angelman

11q24.2-q25	901	5-year-old male with TOF (operated)	Dysmorphism and mental retardation

11q13.4-q14.1	96	4-year-old male with TOF (operated), cleft lip and palate (repaired), broad and bifid nose, small left cornea and eye, GDD, hearing problem, being still unable to suck/drink, long fingers, small philtrum, mental retardation, and so forth	Dysmorphism and mental retardation
