# Supplementary material for: SNP Microarray in FISH Negative Clinically Suspected 22q11.2 Microdeletion Syndrome
Source: Scientifica (Cairo). 2016 Mar 9;2016:5826431. doi: 10.1155/2016/5826431 (PMC4804090; doi:10.1155/2016/5826431)
Supplement: Supplementary file 1 — Clinical proforma for evaluation of suspected 22q11.2 micro-deletion (also provides useful guideline for clinical diagnosis) Supplementary file 2: Master table of the study showing details of FISH and SNP microarray findings of all 101 FISH negative/non-informative samples [file 5826431.f1.zip › 5826431.f1/New folder (2)/6 annexure 1.pdf]

# Proforma (22q11.2 microdeletion syndrome)

Name: Age: Sex: DOB:
Father:
Date (history taking): CR No.: OPD:
Occupation (parents): Education (parents):
Address:
Tel:
Email:

**Pedigree**

Referal Diagnosis:

Chief Complaints:

History:

Examination:
General:

Systemic:

Specific:

| **Column A** | **Column B** | **Column C** |
|---|---|---|
| *The presence of one of the following* | *Two or more of the following core features* | *One core feature plus one of these associated features* |
| Conotruncal cardiac ano-maly such as Fallot's tetralogy, interrupted aortic arch, truncus arteriosus or major aorto-pulmonary collateral arteries | Characteristic facial abnormalities viz. broad bulbous nose, square shaped tip of nose, short filtrum, telecanthus, slant-ing eyes, low set ears, etc | Long slender fingers and hands |
| Parent of an affected child | Non-conotruncal congenital cardiac defect | Short stature |
| | Learning difficulties/deve-lopmental delay | Hypotonia |
| | Cleft palate, velopharyngeal insufficiency or swallowing difficulty | Renal abnormalities or Potter sequence |
| | Hypocalcaemia | Psychiatric (especially bipolar) disorders |
| | Immunodeficiency or thymic hypoplasia | Family history of congenital cardiac defects |

Indications for FISH: Column A (one features); Colummn B (2 or more features); Column C (one column B + one column C features)
